# Comment on Nekkaa et al. *Rhamnus alaternus* Plant: Extraction of Bioactive Fractions and Evaluation of Their Pharmacological and Phytochemical Properties. *Antioxidants* 2021, *10*, 300

**DOI:** 10.3390/antiox12122113

**Published:** 2023-12-14

**Authors:** Alessio Papini

**Affiliations:** CSET/Tropical Herbarium and Department of Biology, University of Florence, Via Micheli 3, 50121 Florence, Italy; alessio.papini@unifi.it; Tel.: +39-055-5275-7399

Analyzing the article “*Rhamnus alaternus* plant: extraction of bioactive fractions and evaluation of their pharmacological and phytochemical properties” by Nekkaa et al. [1] on *Antioxidants*, we report some relevant remarks: we show that, contrarily to the text, *Rhamnus alaternus* has nothing to do with the genus *Reynosia*. Moreover, the intraspecific variation in *R. alathernus* should be considered and, with that, the possible variation in secondary metabolites content. 

I read with interest the useful article “*Rhamnus alaternus* plant: extraction of bioactive fractions and evaluation of their pharmacological and phytochemical properties” by Nekkaa et al. [1] on *Antioxidants*. Despite the general high quality of the article, I noticed that the taxonomic treatment shows some relevant inaccuracies that may affect the value of the scientific results contained in the article.

In Section 2.2 (Botanica Description), the authors state that “*R. alaternus* belongs to the Magnoliophyta division, the Magnoliopsida class, the Rhamnales order, the Rhamnaceae family, the *Reynosia* Genus and the *Rhamnus alaternus* species”.

First of all, *R. alaternus* cannot belong contemporaneously to the genus *Rhamnus* L. and to the genus *Reynosia* Griseb. *Reynosia* is a neotropical genus comprising about 18 species, distributed in the Caribbean region and central America [2], while *Rhamnus* is a large paleotropical and temperate genus comprising about 200 species of the Northern hemisphere [3]. Even if genera *Rhamnus* and *Reynosia* belong to the same tribe (Rhamneae), the secretory structures are not always the same, with idioblasts containing mucilage mainly in the flower being the most common situation in *Reynosia*, and idioblasts and other structures localized in the leaf and containing tannins in *Rhamnus* [4], with evident consequences about their content in bioactive compounds.

The second point is the intraspecific tratment of *Rhamnus alaternus* by Nekkaa et al. [1]: “In addition, this plant has various synonyms including *R. alaternus* var. *angustifolia* DC, *R. alaternus* var. *balearica* DC, *R. alaternus* var. *hispanica* DC, and *R. alaternus* var. *vulgaris* DC”. Technically, the listed varieties are not synonyms of *Rhamnus alaternus*, but rather a representation of an unrecognized infraspecific biodiversity at the variety level. Could it be relevant for the investigation of the bioactive properties of *Rhamnus alaternus*? The answer is positive, since in *Rhamnus alaternus*, at least five subspecies are recognized and they may partially overlap with old varieties by De Candolle and other subspecific entities of uncertain relationships with *R. alaternus* s. str., included in section *Alaternus* Grubov of genus *Rhamnus* [5].

I propose here these simple corrections together with an important question: how much taxonomic inaccuracy may affect the results about pharmaceutical properties of plants? The collaboration between taxonomists and experts in phytochemistry should probably increase, in order to better explore how the systematic biodiversity might influence the biodiversity in secondary metabolites in plants.
